# Prognostic Factors and Survival in Primary Central Nervous System Lymphoma: A Population-Based Study

**DOI:** 10.1155/2018/7860494

**Published:** 2018-06-28

**Authors:** Yudong Shan, Yilan Hu

**Affiliations:** Department of Neurosurgery, Affiliated Cixi Hospital of Wenzhou Medical University, Ningbo, Zhejiang Province, China

## Abstract

**Objective:**

This study sought to explore the prognostic factors in a large retrospective cohort of patients with primary central nervous system lymphoma (PCNSL) from the Surveillance, Epidemiology, and End Results database.

**Methods:**

There were 5903 patients with PCNSL who had complete clinical information and were identified in the Surveillance, Epidemiology, and End Results program between 1973 and 2014. The epidemiology, therapeutic measures, and clinical characteristics were listed as descriptive statistics. They were grouped into 4 categories: immunocompetent individual with diffuse large B cell lymphoma (DLBCL), immunocompetent individual with non-DLBCL, immunocompromised individual with DLBCL, and immunocompromised individual with non-DLBCL based on different subtypes and immunological status. Survival analysis was conducted with Cox regression models.

**Results:**

Different demographics and clinical characteristics were identified as independent factors in different groups. In survival analysis, for patients with DLBCL, chemotherapy involving treatments was associated with the most favorable survival. Received-only radiation could be considered as a primary treatment in immunocompetent patients with non-DLBCL. These differences were statistically significant (*P* < 0.05).

**Conclusion:**

PCNSL patients treated with appropriate chemotherapy treatments may receive stable tumor control.

## 1. Introduction

Primary central nervous system lymphoma (PCNSL) is a rare type of extranodal non-Hodgkin's lymphoma (NHL), which is a rare and heterogeneous disease that accounts for approximately 1–3% of all central nervous system (CNS) tumors [1]. The estimated annual incidence of PCNSL, as calculated by the rate session of SEER statistics, is 0.7 cases per 100,000 person-years in the United States. It is well known that PCNSL occurs more commonly in immunosuppressed populations, particularly among people with human immunodeficiency virus (HIV) infection. However, an increasing incidence among immunocompetent adults requires more attention. Improved diagnosis with ascertainment bias and increasing health awareness may explain this situation [2, 3]. Different etiologies appear in immunocompetent and immunosuppressed individuals. Epstein-Barr Virus (EBV) is the major cause of PCNSL in HIV-infected people and transplant recipients, but it is less common in immunocompetent individuals [4, 5]. The heterogeneity of the disease leads to different clinical outcomes and diverse therapeutic approaches.

For decades, radiation and chemotherapy have gained wide recognition as the main treatments for cancer. Cancer-directed surgical procedures have become an unorthodox treatment [6, 7]. Unfortunately, high rates of disease control or cures have not been proven by the sensitivity of radiation and chemotherapy. Traditionally, whole brain radiation therapy (WBRT) was the primary treatment option, but currently, physicians rarely use radiation alone as the primary treatment. Regardless of whether these treatments are performed alone or in combination, high rates of neurotoxicity, particularly in patients over 60 years of age, should be considered [8]. Some studies have indicated that the combination of radiation with chemotherapy was better than a single treatment, whereas another study demonstrated that radiation should not be included in combinations [9, 10]. The prognostic role of radiation in treating PCNSL remains controversial. Therefore, in this study, we intend to explore the prognostic factors for survival in patients with PCNSL from the National Cancer Institute's Surveillance, Epidemiology, and End Results (SEER) database.

## 2. Materials and Methods

### 2.1. Data Source

The data source for this study is the SEER database from 1973 to 2014, which was released in November 2016. The SEER program provides clinical data, such as patient demographics and tumor characteristics, annually and openly. The National Center for Health Statistics is responsible for mortality data collection and updates [11]. The necessary data were obtained by the National Cancer Institute's SEER^∗^Stat software (Surveillance Research Program, National Cancer Institute SEER^∗^Stat software, http://www.seer.cancer.gov/seerstat) (Version 8.3.4).

### 2.2. Inclusion Criteria

The inclusion criteria for the study were as follows: patients who were diagnosed between 1973 and 2014 with complete clinical manifestations, patients who had CNS listed as the primary disease lesion (International Classification of Diseases for Oncology, 3rd Edition (ICD-O-3) topography codes C70.0–C72.9), and patients who were diagnosed as having NHL subtypes (ICD-O-3 histology codes 9590, 9591, 9670–9699, 9701–9705, 9714, 9719, and 9727–9729). All diagnoses were histologically confirmed. All patients included were regularly followed up. Immunological status was not provided directly in the SEER database, and it was presumed by cause of death. Thus, on some level, “uncertain criteria” were included in the inclusion criteria. Patients with an insufficient clinical profile, unknown cause of death, and unknown survival months were excluded. Finally, 5903 patients were enrolled. This study was conducted based on the Public Data Base, and all methods were carried out in accordance with the Declaration of Helsinki. No experiments on humans or the use of human tissue samples were used in this study.

### 2.3. Variables for the Analyses

Age at diagnosis, sex, race, lymphoma subtypes, tumor primary sites, tumor laterality, lymphoma stage, therapy modality, immunological status, insurance status, marital status, and overall survival (OS) were extracted from original data and considered primary variables. Age at diagnosis was categorized as less than 60 years and 60 years or older. Race was classified into African American, non-Hispanic Caucasian, and others. Lymphoma subtypes were dichotomized as diffuse large B cell lymphoma (DLBCL) and non-DLBCL. Primary sites were located in the brain, meninges/cranial nerves, spine, and other locations. Tumor laterality was defined as a binary variable, with a nonpaired site and a paired site (left/right—origin of primary). According to SEER research data record description, tumor laterality describes the side of a paired organ or side of the body on which the reportable tumor originated. Laterality is coded for select invasive, benign, and borderline primary intracranial and CNS tumors. Lymphoma stage was stratified according to the Ann Arbor stage [12, 13]. The therapeutic modality included surgery, radiation, and chemotherapy. Detailed surgical information was not reviewed because its role in PCNSL was mainly limited to biopsy for tissue diagnosis. The immunological status was not provided directly in the SEER database. However, the cause of death was available. One of these causes was defined as “Other Infectious and Parasitic Diseases, including HIV.” Patients with this cause were strongly assumed to be immunocompromised. Further information involving HIV-positive, transplantation, or other causes were not supplied by SEER Database. This is similar to the approach taken by previous studies [14, 15]. Patients living with an immunocompromised status were rather difficult to identify by the records. However, this status had a limited impact on the total conclusion because patients with this status did not have an absolute predominance in terms of quantity. Insurance status was defined as uninsured, insured, or any Medicaid and insurance status unknown. Marital status was categorized as never married, married, ever married (including divorce, separated, and widowed), and marital status unknown.

### 2.4. Outcome Measurement

OS was the observed indicator of outcomes. OS was determined by the “vital status,” which represents the time from the date of diagnosis to the date of death. Death was considered a separate event. Censored observations included patients who were still alive at the time of the last follow-up. We only performed analysis on OS because we found that the results for cancer-specific survival were the same as those obtained for OS in the univariate and multivariate analyses.

### 2.5. Statistical Analysis

All statistical analyses were calculated by Statistical Package for the Social Sciences (SPSS) software version 22 (SPSS Inc., Chicago, IL, USA). Univariate and multivariate Cox proportional hazard models were used to estimate the association between various covariates and survival outcome. Kaplan-Meier curves and the log-rank (Mantel-Cox) test were used to compare the OS rates. Differences were considered statistically significant when *P* < 0.05.

## 3. Results

### 3.1. Baseline Characteristics

A total of 3353 males and 2550 female patients were included in the analysis. Of the total, 5138 patients were considered immunocompetent, and 765 were immunocompromised. The majority of them (4685, 79.4%) were diagnosed between 1996 and 2014. The median age at diagnosis was 61 years. Patients with ages ranging from 0 years to 98 years (Q25 47 years, Q75 71 years) were analyzed, in which patients with ages ≥ 60 years were the most common (53.7%). The immunocompromised cohort was substantially younger than the immunocompetent cohort was (for patients ≥ 60, 3.7% versus 61.1%). More blacks (28.2%) and males (88.9%) were included in the immunocompromised cohort than in the immunocompetent cohort (6% and 52%, resp.). DLBCL was the primary subtype in both cohorts. The tumor was usually located in the brain as the primary lesion. More azygous lesions than paired lesions were detected in patients. Patients had a larger proportion stratified as Ann Arbor stages I–II. More patients in the immunocompromised cohort (60.8%) underwent radiation than did those in the immunocompetent cohort (45.2%). In contrast, the patients in the immunocompetent cohort were more likely to receive chemotherapy than were those in the immunocompromised cohort (61.9% versus 19.2%). The insurance status of most patients was unknown. Most immunocompromised patients never got married (74%), while most immunocompetent patients were or ever got married. For immunocompromised patients, radiation only (49.3%) and neither but conservative treatment (30.5%) were the main therapeutic approaches. In contrast, most immunocompetent patients received chemotherapy treatments. The demographics and clinical characteristics are summarized in Table 1.

### 3.2. Univariate and Multivariate Analyses in Patients according to Lymphoma Subtypes and Immunological Status

The variables that were validated as independent prognostic factors in immunocompromised patients with DLBCL included Ann Arbor stage (stages III–IV, hazard ratio (HR): 1.499, 95% CI: 1.187–1.892, *P* = 0.001), received radiation therapy (yes, HR: 0.662, 95% CI: 0.548–0.800, *P* < 0.001), and received chemotherapy (yes, HR: 0.554, 95% CI: 0.434–0.709, *P* < 0.001) (Table 2). The independent prognostic factors in immunocompetent patients with DLBCL included age (range of over 60 years, HR: 1.834, 95% CI: 1.679–2.004, *P* < 0.001), primary site (primary lesion of meninges/cranial nerves, HR: 0.470, 95% CI: 0.313–0.706, *P* < 0.001; primary lesion of spine, HR: 0.428, 95% CI: 0.342–0.535, *P* < 0.001; and other primary lesion, HR: 0.715, 95% CI: 0.620–0.824, *P* < 0.001), tumor laterality (paired primary site, HR: 0.905, 95% CI: 0.825–0.994, *P* = 0.036), received chemotherapy (yes, HR: 0.442, 95% CI: 0.407–0.480, *P* < 0.001), and marital status (ever married, HR: 1.354 95% CI: 1.183–1.549, *P* < 0.001) (Table 3). Received chemotherapy (HR: 0.384, 95% CI: 0.273–0.539, *P* < 0.001) was the only risk factor in immunocompromised patients with non-DLBCL (Table 4). In immunocompetent patients with non-DLBCL, age (range of over 60 years, HR: 2.389, 95% CI: 2.052–2.780, *P* < 0.001), primary site (primary lesion of meninges/cranial nerves, HR: 0.349, 95% CI: 0.238–0.511, *P* < 0.001; primary lesion of spine, HR: 0.452, 95% CI: 0.362–0.565, *P* < 0.001; and other primary lesion, HR: 0.515, 95% CI: 0.428–0.618, *P* < 0.001), received chemotherapy (yes, HR: 0.724, 95% CI: 0.433–0.828, *P* < 0.001), and marital status (married, HR: 0.821 95% CI: 0.677–0.994, *P* = 0.043) were identified as risk factors (Table 5). These results were calculated based on multivariate analysis.

### 3.3. Impact of Treatment Options for Patients with DLBCL

**Figure 1:**
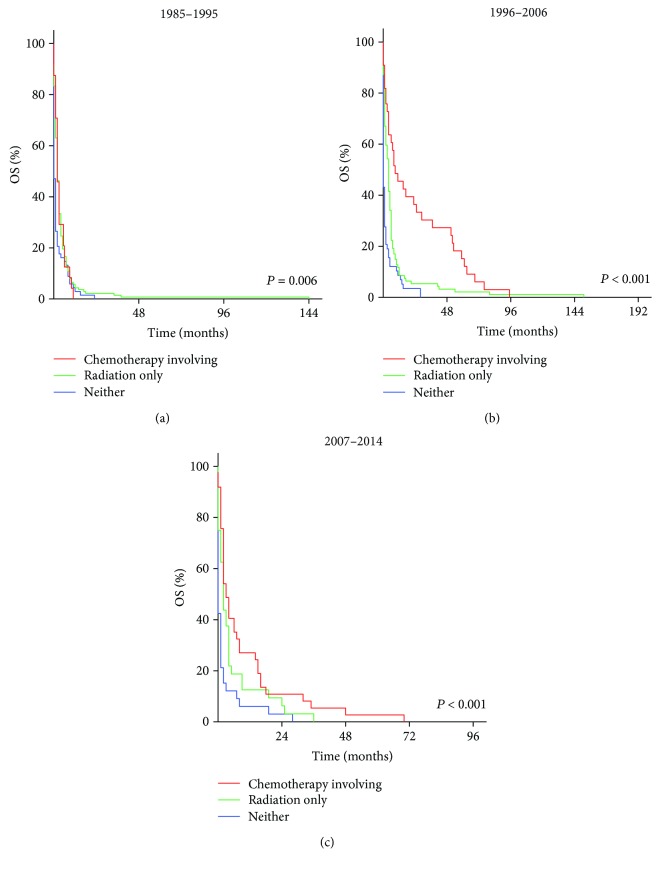
Kaplan-Meier survival curves of immunocompromised patients with diffuse large B cell lymphoma (DLBCL) in 1985–1995 (a), 1996–2006 (b), and 2007–2014 (c) according to treatment options.

**Figure 2:**
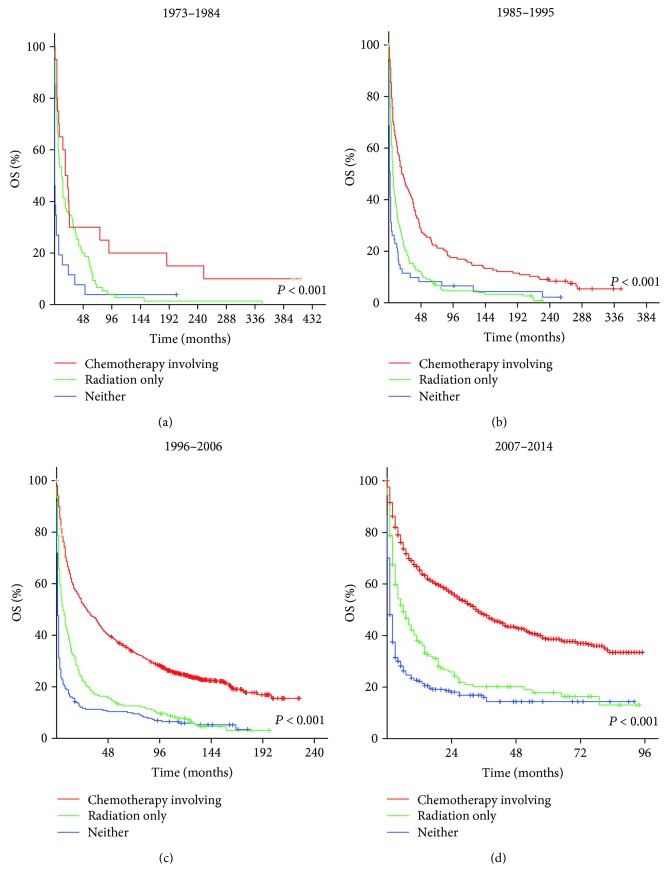
Kaplan-Meier survival curves of immunocompetent patients with DLBCL in 1973–1984 (a), 1985–1995 (b), 1996–2006 (c), and 2007–2014 (d) according to treatment options.

To compare the efficacy of different treatments, we grouped them as follows: received radiation only, received chemotherapy treatments (chemotherapy only or chemotherapy plus radiation), and received neither. In many countries, PCNSL patients first undergo chemotherapy. When the therapeutic response is good, the patients may be followed up without radiotherapy, but when complete remission is not obtained or the tumor recurs, radiation therapy may be added. Patients who receive chemotherapy plus radiation may thus have an apparently worse background than those who receive chemotherapy alone. Therefore, it is not appropriate to separate these two groups. Stratification analysis made the results more accurate. Survival analysis beyond different treatments was conducted according to different immunological statuses and dates of diagnosis. For immunocompromised patients, the median OS for those who received radiation only was 2 months (1985–1995), 4 months (1996–2006), and 2 months (2007–2014), respectively. The median OS for those who received neither was 0 months (1985–1995, 1996–2006, and 2007–2014). The median OS for those who received chemotherapy involving treatments was 2 months (1985–1995), 9 months (1996–2006), and 3 months (2007–2014). For immunocompetent patients, the median OS for those who received radiation only was 12 months (1973–1984), 6 months (1985–1995), 6 months (1996–2006), and 2 months (2007–2014). The median OS for those received neither was 0 months (1973–1984), 2 months (1985–1995), 1 month (1996–2006), and 1 month (2007–2014). The median OS for those who received chemotherapy treatments was 18 months (1973–1984), 20 months (1985–1995), 28 months (1996–2006), and 34 months (2007–2014). These differences were statistically significant (*P* < 0.01). The Kaplan-Meier-estimated OS distributions are shown in Figures 1 and 2.

### 3.4. Impact of Treatment Options for Patients with Non-DLBCL

**Figure 3:**
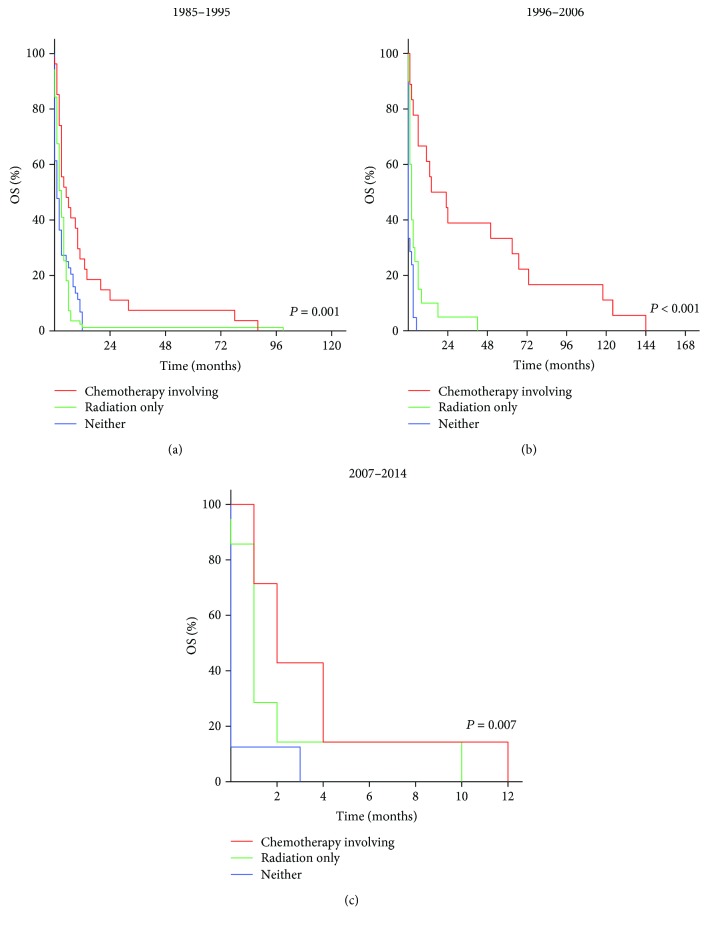
Kaplan-Meier survival curves of immunocompromised patients with non-DLBCL in 1985–1995 (a), 1996–2006 (b), and 2007–2014 (c) according to treatment options.

**Figure 4:**
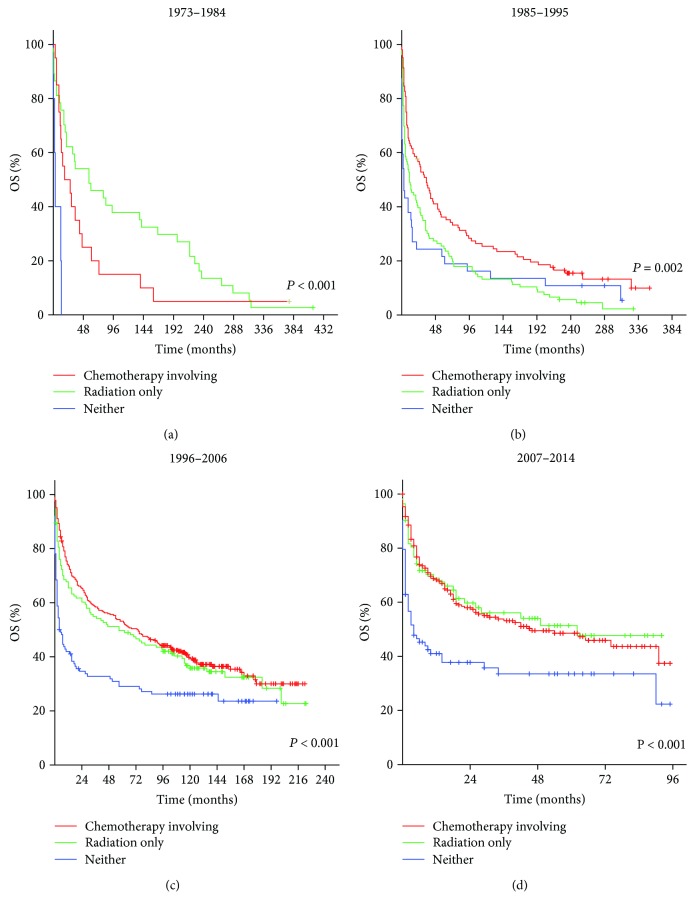
Kaplan-Meier survival curves of immunocompetent patients with non-DLBCL in 1973–1984 (a), 1985–1995 (b), 1996–2006 (c), and 2007–2014 (d) according to treatment options.

**Figure 5:**
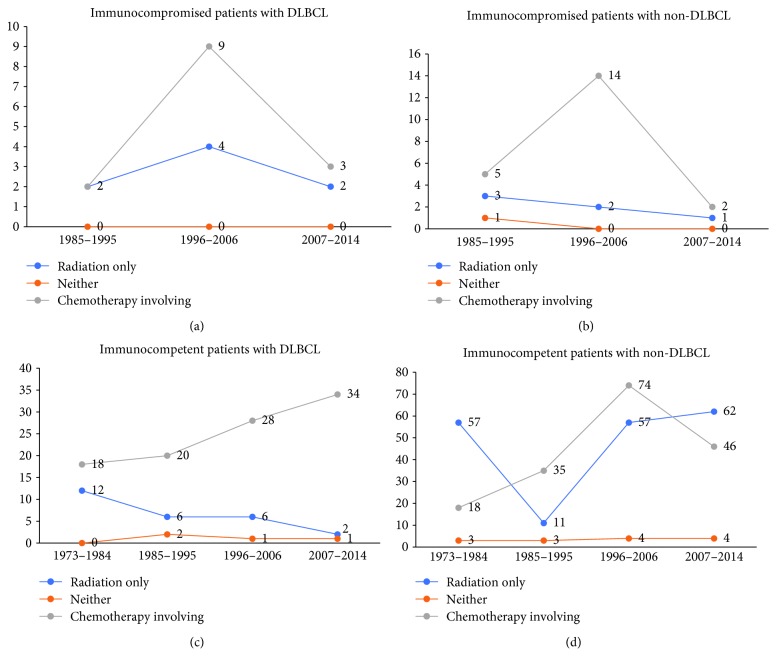
The median OS (months) of each group of patients in any periods are displayed using line charts: (a) immunocompromised patients with DLBCL, (b) immunocompromised patients with non-DLBCL, (c) immunocompetent patients with DLBCL, and (d) immunocompetent patients with non-DLBCL

For immunocompromised patients, the median OS for those who received radiation only was 3 months (1985–1995), 2 months (1996–2006), and 1 month (2007–2014). The median OS for those who received neither was 1 month (1985–1995), 0 months (1996–2006), and 0 months (2007–2014). The median OS for those who received chemotherapy treatments was 5 months (1985–1995), 14 months (1996–2006), and 2 months (2007–2014). For immunocompetent patients, the median OS for those who received radiation only was 57 months (1973–1984), 11 months (1985–1995), 57 months (1996–2006), and 62 months (2007–2014). The median OS for those who received neither was 3 months (1973–1984), 3 months (1985–1995), 4 months (1996–2006), and 4 months (2007–2014). The median OS for those who received chemotherapy treatments was 18 months (1973–1984), 35 months (1985–1995), 74 months (1996–2006), and 46 months (2007–2014). These differences were statistically significant (*P* < 0.01). The Kaplan-Meier-estimated OS distributions are shown in Figures 3 and 4. The median OS of each group in each period was summarized and are shown in Figure 5.

## 4. Discussion

Previous studies focused mostly on either immunocompetent patients or the impact of demographic factors [14, 16–18]. Chemotherapy data were first available in population-based data collected in the United States in 2017, which made it possible to analyze the merits and demerits of diverse therapeutic approaches for PCNSL to demonstrate an improvement in survival over time. Some predictors of survival, such as age, immunological status, and gender, have been confirmed in this study. However, some well-known predictors have been challenged. Receiving radiation seemed to be beneficial only for survival, which contradicts Norden et al.'s conclusion [14] based on the SEER database up to 2004. Being married at diagnosis did not have a favorable impact on survival compared to being unmarried, but ever being married (including divorce, separated, and widowed) actually negatively influenced survival in patients with DLBCL. Survival over time has improved for years and has mainly been attributed to the increasing use of methotrexate-based chemotherapy regimens and improved supportive care. The standard therapeutic approaches for incipient patients or relapsed or refractory patients are a high-dose methotrexate-based regimen or (low-dose or standard-dose) WBRT according to the NCCN Clinical Practice Guidelines in Oncology: Central Nervous System Cancers Version 1.2016. However, several patients who received radiation therapy showed a decreased survival time [15, 19, 20]. Presumably physicians and elderly patients, who represent approximately half of the PCNSL population, are aware of the fact that radiation therapy is neurotoxic. This change in benefit affects physicians' decisions. Here, we sought to retrospectively identify evidence about the impact of radiation on survival from a population-based database.

In immunocompromised patients, regardless of whether they have been diagnosed with DLBCL or non-DLBCL, chemotherapy treatments could prolong survival in all time periods. It is better to choose this as the primary treatment. Additionally, some benefit could be observed in patients who received radiation only. Without these two approaches, immunocompromised patients have little chance of survival. It is obvious that the immunocompromised patients who received neither chemotherapy nor radiation had a median OS of 0 months. How could this occur? Detailed analysis showed that this population of patients was mainly male (possibly homosexual), black, single, young, or having poor economic and education conditions, which may prevent them from receiving further treatment. Furthermore, faint medical awareness, which can lead to a long duration before seeking medical assistance and concerns about basic immunodeficiency diseases (almost HIV), from the physician may also be a cause. This type of patient unexpectedly represents one-third of all immunocompromised patients, and it has been a general social phenomenon in the US. The results became more interesting in immunocompetent patients. Chemotherapy treatments provide more benefits over time in DLBCL patients, which is mainly attributed to the improvement of chemotherapy drugs and regimens. Only radiation therapy is below the therapeutic need and may cause central neurotoxicity. The relevant survival time is relatively shorter than that in those who received chemotherapy treatments in recent years. However, the results were the reverse in non-DLBCL patients. Although great advancements have been made in chemotherapy treatments, it was still seen that the efficacy of radiation therapy was not inferior to that of chemotherapy treatments and became even better in the last 7 years. It is well known that non-DLBCL has a less aggressive evolution than DLBCL does, so less radical therapeutic approaches may be associated with favorable survival. In any case, receiving neither chemotherapy nor radiation is not recommended. We observed that most patients were at Ann Arbor stages I–II, which is considered an early stage of NHL, but only less than 30% cases survived, regardless of treatment. There is still a long way to go in cancer control.

Previous studies have collected relevant results based on a limited population. Chalise et al. [21] indicated that a dexamethasone-based chemotherapy regimen is comparable with that of an HD-MTX-based chemotherapy regimen plus WBRT. Korfel et al. suggested no worsening of OS without WBRT in the primary therapy of PCNSL, even although statistical proof of noninferiority in OS was not given [22]. Thiel et al. showed that no significant difference in OS was recorded when WBRT was omitted from first-line chemotherapy in patients with newly diagnosed primary CNS lymphoma [10]. Our result further consolidates their conclusion. It actually does not mean that radiation could be neglected. Dose-reduced WBRT or partial-brain radiotherapy has been studied and evaluated. Early results have shown their potential [23, 24]. Even the role of surgery as an unorthodox treatment should be reconsidered [25].

The latest data and the relatively large cohort are the major strengths of this study, which closely represents the current situation in the US. Conclusions about mortality and the outcomes of rare malignancies could be found only in studies of large populations but not for collaborative or single institutions. However, the known limitations of the study may weaken these conclusions. Our effort to identify immunocompromised patients has inherent flaws, although there are no better methods. In addition, this classification scheme relies primarily on the causes of death. A few surviving immunocompromised patients might be classified as immunocompetent. The inability to accurately classify immunocompromised patients in the SEER data has the potential to introduce bias. However, this situation cannot be solved until a marker of immunological status, such as HIV and transplantation status, is clearly labelled in the SEER database. In addition, detailed information about chemotherapy and radiation was not captured. This information is not currently available from the SEER database.

In summary, these data indicate that radiation alone should not be recommended as the primary therapeutic approach for PCNSL patients with the DLBCL subtype. In non-DLBCL cases, it may provide an excellent outcome. Receiving neither chemotherapy nor radiation led to poor survival. The use of proper chemotherapy regimens greatly improved survival over time. Novel adjuvant therapies should be developed.

## Figures and Tables

**Table 1:** Summary of characteristics for the patient population. SEER 1973–2014 (*n* = 5903)^a^.

Characteristic	All patients number (%) (*n* = 5903)	Immunocompromised patients number (%) (*n* = 765)	Immunocompetent patients number (%) (*n* = 5138)
Age			
<60	2735 (46.3)	737 (96.3)	1998 (38.9)
≥60	3168 (53.7)	28 (3.7)	3140 (61.1)
Sex			
Male	3353 (56.8)	680 (88.9)	2673 (52.0)
Female	2550 (43.2)	85 (11.1)	2465 (48.0)
Race			
Black	523 (8.9)	216 (28.3)	307 (6.0)
White	4816 (81.5)	525 (68.6)	4291 (83.5)
Other (American Indian/AK native, Asian/Pacific Islander)	564 (9.6)	24 (3.1)	540 (10.5)
Dates of diagnosis			
1973–1984	188 (3.2)	5 (0.7)	183 (3.6)
1985–1995	1030 (17.4)	386 (50.5)	644 (12.5)
1996–2006	2265 (38.4)	250 (32.7)	2015 (39.2)
2007–2014	2420 (41.0)	124 (16.1)	2296 (44.7)
Lymphoma subtypes			
DLBCL	4342 (73.6)	527 (68.9)	3815 (74.3)
Non-DLBCL	1561 (26.4)	238 (31.1)	1323 (25.7)
Primary site			
Brain	4768 (80.8)	684 (89.4)	4084 (79.5)
Meninges/cranial nerves	110 (1.9)	7 (0.9)	107 (2.0)
Spine	364 (6.1)	18 (2.4)	346 (6.7)
Other	661 (11.2)	56 (7.3)	605 (11.8)
Laterality			
Not a paired site	4197 (71.7)	662 (86.5)	3535 (68.8)
Paired site (left/right—origin of primary)	1706 (28.9)	103 (13.5)	1603 (31.2)
Lymphoma Ann Arbor stage			
I–II	4324 (73.3)	585 (76.5)	3739 (72.8)
III–IV	116 (19.6)	147 (19.2)	1015 (19.8)
Unknown	417 (7.1)	33 (4.3)	384 (7.5)
Radiation			
No	3015 (51.1)	289 (37.8)	2726 (53.1)
Yes	2785 (47.2)	465 (60.8)	2320 (45.2)
Unknown	103 (1.7)	11 (1.4)	92 (1.8)
Chemotherapy			
Yes	3325 (56.3)	147 (19.2)	3178 (61.9)
No	2578 (43.7)	618 (80.8)	1960 (38.1)
Immunological status			
Immunocompromised	765 (13.0)	—	—
Immunocompetent	5138 (87.0)		
Vital status			
Dead	4382 (74.2)	765 (100.0)	3617 (70.4)
Alive	1521 (25.8)	0 (0.0)	1521 (29.6)
Insurance status			
Uninsured	101 (1.7)	12 (1.6)	89 (1.7)
Insured/any Medicaid	2271 (38.5)	107 (14.0)	2164 (42.1)
Insurance status unknown	3531 (59.8)	646 (84.4)	2885 (56.2)
Marital status			
Never married	1409 (23.9)	566 (74.0)	843 (16.4)
Married	3170 (53.7)	107 (14.0)	3063 (59.6)
Ever married (divorced, separated, and widowed)	1142 (19.3)	63 (8.2)	1079 (21.0)
Unknown	182 (3.1)	9 (3.8)	153 (3.0)
Multiple modalities			
Neither but conservative treatment	1104 (18.7)	233 (30.5)	871 (17.0)
Chemotherapy involving	3270 (55.4)	144 (18.8)	3126 (60.8)
Only radiation	1426 (24.2)	377 (49.3)	1049 (20.4)
Missing	103 (1.7)	11 (1.4)	92 (1.8)

SEER: Surveillance, Epidemiology, and End Results; DLBCL: diffuse large B cell lymphoma; PCNSL: primary central nervous system lymphoma. ^a^Data are presented as the number (percentage) of patients.

**Table 2:** Univariate and multivariate survival analysis in immunocompromised patients with DLBCL.

Variable	Univariate analysis		Multivariate analysis	
	HR (95% CI)	*P*	HR (95% CI)	*P*
Age (years)				
<60	Reference			
≥60	0.727 (0.453–1.166)	0.186		
Race				
White	Reference			
Black	1.194 (0.988–1.443)	0.067		
Other (American Indian/AK native, Asian/Pacific Islander)	0.921 (0.573–1.480)	0.734		
Gender				
Female	Reference			
Male	1.185 (0.908–1.546)	0.212		
Dates of diagnosis				
1973–1984	Reference		Reference	
1985–1995	3.253 (1.027–10.304)	0.045	2.701 (0.472–15.466)	0.264
1996–2006	2.270 (0.718–7.179)	0.163	1.881 (0.329–10.747)	0.477
2007–2014	2.740 (0.858–8.756)	0.089	2.376 (0.411–13.728)	0.334
Primary site				
Brain	Reference		Reference	
Meninges/cranial nerves	0.313 (0.076–1.287)	0.107	0.603 (0.073–5.004)	0.640
Spine	0.465 (0.254–0.852)	0.013	0.487 (0.262–0.906)	0.023
Other	0.593 (0.709–1.541)	0.824	1.052 (0.692–1.600)	0.811
Laterality				
Not a paired site	Reference			
Paired site (left/right—origin of primary)	0.901 (0.709–1.145)	0.393		
Lymphoma Ann Arbor stage				
I–II	Reference		Reference	
III–IV	1.452 (1.157–1.824)	0.001	1.499 (1.187–1.892)	0.001
Unknown	1.269 (0.833–1.935)	0.267	1.177 (0.735–1.886)	0.497
Radiation				
No	Reference		Reference	<0.001
Yes	0.673 (0.562–0.806)	<0.001 0.569	0.662 (0.548–0.800)	0.925
Unknown	0.823 (0.422–1.607)		0.968 (0.490–1.914)	
Chemotherapy				
No	Reference		Reference	
Yes	0.585 (0.465–0.735)	<0.001	0.554 (0.434–0.709)	<0.001
Insurance status				
Uninsured	Reference			
Insured/any Medicaid	0.708 (0.378–1.328)	0.282		
Insurance status unknown	0.735 (0.403–1.339)	0.315		
Marital status				
Never married	Reference			
Married	0.847 (0.660–1.087)	0.192		
Ever married (divorced, separated, and widowed)	0.877 (0.647–1.189)	0.399		
Unknown	1.097 (0.731–1.646)	0.654		

OS: overall survival; HR: hazard ratio; CI: confidence interval; SEER: Surveillance, Epidemiology, and End Results; DLBCL: diffuse large B cell lymphoma; PCNSL: primary central nervous system lymphoma.

**Table 3:** Univariate and multivariate survival analysis in immunocompetent patients with DLBCL.

Variable	Univariate analysis		Multivariate analysis	
	HR (95% CI)	*P*	HR (95% CI)	*P*
Age (years)				
<60	Reference		Reference	
≥60	2.084 (1.916–2.267)	<0.001	1.834 (1.679–2.004)	<0.001
Race				
White	Reference		Reference	
Black	0.759 (0.628–0.918)	0.004	0.854 (0.703–1.037)	0.111
Other (American Indian/AK native, Asian/Pacific Islander)	0.890 (0.788–1.006)	0.062	0.912 (0.806–1.031)	0.140
Gender				
Female	Reference			
Male	0.947 (0.878–1.021)	0.153		
Dates of diagnosis				
1973–1984	Reference		Reference	
1985–1995	1.937 (0.761–1.153)	0.536	1.124 (0.912–1.386)	0.272
1996–2006	0.727 (0.600–0.879)	0.001	1.048 (0.861–1.275)	0.642
2007–2014	0.636 (0.525–0.770)	<0.001	1.031 (0.841–1.265)	0.767
Primary site				
Brain	Reference		Reference	
Meninges/cranial nerves	0.467 (0.312–0.698)	<0.001	0.470 (0.313–0.706)	<0.001
Spine	0.403 (0.323--0.503)	<0.001	0.428 (0.342–0.535)	<0.001
Other	0.682 (0.594–0.783)	<0.001	0.715 (0.620–0.824)	<0.001
Laterality				
Not a paired site	Reference		Reference	
Paired site (left/right—origin of primary)	0.885 (0.815–0.961)	0.003	0.905 (0.825–0.994)	0.036
Lymphoma Ann Arbor stage				
I–II	Reference			
III–IV	0.967 (0.877–1.067)	0.504		
Unknown	1.254 (1.073–1.465)	0.004		
Radiation				
No	Reference			
Yes	1.038 (0.962–1.120)	0.338		
Unknown	0.815 (0.615–1.081)	0.156		
Chemotherapy				
No	Reference		Reference	
Yes	0.402 (0.372–0.434)	<0.001	0.442 (0.407–0.480)	<0.001
Insurance status				
Uninsured	Reference			
Insured/any Medicaid	0.880 (0.646–1.198)	0.416		
Insurance status unknown	1.090 (0.802–1.481)	0.581		
Marital status				
Never married	Reference		Reference	
Married	1.254 (1.118–1.406)	<0.001	1.070 (0.949–1.207)	0.271< 0.001 0.472
Ever married (divorced, separated, and widowed)	1.823 (1.603–2.074)	<0.001	1.354 (1.183–1.549)	
Unknown	1.632 (1.283–2.075)	<0.001	1.093 (0.857–1.394)	

OS: overall survival; HR: hazard ratio; CI: confidence interval; SEER: Surveillance, Epidemiology, and End Results; DLBCL: diffuse large B cell lymphoma; PCNSL: primary central nervous system lymphoma.

**Table 4:** Univariate and multivariate survival analysis in immunocompromised patients with non-DLBCL.

Variable	Univariate analysis		Multivariate analysis	
	HR (95% CI)	*P*	HR (95% CI)	*P*
Age (years)				
<60	Reference			
≥60	0.599 (0.305–1.176)	0.136		
Race				
White	Reference			
Black	1.029 (0.766–1.382)	0.848		
Other (American Indian/AK native, Asian/Pacific Islander)	2.129 (0.936–4.841)	0.071		
Gender				
Female	Reference			
Male	0.874 (0.568–1.346)	0.541		
Dates of diagnosis				
1973–1984	Reference			
1985–1995	1.396 (0.344–5.661)	0.641		
1996–2006	1.056 (0.257–4.347)	0.939		
2007–2014	2.218 (0.518–9.505)	0.283		
Primary site				
Brain	Reference			
Meninges/cranial nerves	1.068 (0.439–2.598)	0.885		
Spine	0.784 (0.363–1.690) 0.620	0.534		
Other	(0.414–0.929)	0.021		
Laterality				
Not a paired site	Reference			
Paired site (left/right—origin of primary)	1.383 (0.905–2.115)	0.134		
Lymphoma Ann Arbor stage				
I–II	Reference			
III–IV	0.968 (0.708–1.322)	0.836		
Unknown	0.938 (0.495–1.779)	0.846		
Radiation				
No	Reference			
Yes	0.803 (0.617–1.046)	0.104		
Unknown	0.454 (0.111–1.846)	0.270		
Chemotherapy				
No	Reference		Reference	
Yes	0.384 (0.273–0.539)	<0.001	0.384 (0.273–0.539)	<0.001
Insurance status				
Uninsured	Reference			
Insured/any Medicaid	0.349 (0.046–2.631)	0.307		
Insurance status unknown	0.222 (0.031–1.605)	0.136		
Marital status				
Never married	Reference			
Married	0.635 (0.430–0.936)	0.022		
Ever married (divorced, separated, and widowed)	0.885 (0.531–1.477)	0.641		
Unknown	2.138 (0.788–5.799)	0.136		

OS: overall survival; HR: hazard ratio; CI: confidence interval; SEER: Surveillance, Epidemiology, and End Results; DLBCL: diffuse large B cell lymphoma; PCNSL: primary central nervous system lymphoma.

**Table 5:** Univariate and multivariate survival analysis in immunocompetent patients with non-DLBCL.

Variable	Univariate analysis		Multivariate analysis	
	HR (95% CI)	*P*	HR (95% CI)	*P*
Age (years)				
<60	Reference		Reference	
≥60	2.399 (2.085–2.759)	<0.001	2.389 (2.052–2.780)	<0.001
Race				
White	Reference			
Black	0.777 (0.608–0.993)	0.043		
Other (American Indian/AK native, Asian/Pacific Islander)	1.016 (0.793–1.300)	0.902		
Gender				
Female	Reference	0.946		
Male	1.005 (0.880–1.147)			
Dates of diagnosis				
1973–1984	Reference			
1985–1995	1.165 (0.876–1.549)	0.294		
1996–2006	0.761 (0.577–1.002)	0.052		
2007–2014	0.847 (0.633–1.135)	0.266		
Primary Site				
Brain	Reference		Reference	
Meninges/cranial nerves	0.358 (0.245–0.524)	<0.001	0.349 (0.238–0.511)	<0.001
Spine	0.443 (0.355--0.553)	<0.001	0.452 (0.362–0.565)	<0.001
Other	0.555 (0.463–0.666)	<0.001	0.515 (0.428–0.618)	<0.001
Laterality				
Not a paired site	Reference			
Paired site (left/right—origin of primary)	0.962 (0.804–1.150)	0.670		
Lymphoma Ann Arbor stage				
I–II	Reference			
III–IV	0.980 (0.835–1.152)	0.810		
Unknown	1.008 (0.832–1.220)	0.936		
Radiation				
No	Reference			
Yes	0.894 (0.780–1.023)	0.104		
Unknown	1.978 (1.198–3.267)	0.008		
Chemotherapy				
No	Reference		Reference	
Yes	0.684 (0.599–0.781)	<0.001	0.724 (0.633–0.828)	<0.001
Insurance status				
Uninsured	Reference			
Insured/any Medicaid	1.457 (0.685–3.096)	0.328		
Insurance status unknown	1.522 (0.722–3.208)	0.270		
Marital status				
Never married	Reference		Reference	
Married	1.153 (0.961–1.383)	0.126	0.821 (0.677–0.994)	0.043
Ever married (divorced, separated, and widowed)	1.689 (1.363–2.094)	<0.001	1.048 (0.833–1.319)	0.689
Unknown	1.206 (0.805–1.805	0.364	0.809 (0.538–1.217)	0.309

OS: overall survival; HR: hazard ratio; CI: confidence interval; SEER: Surveillance, Epidemiology, and End Results; DLBCL: diffuse large B cell lymphoma; PCNSL: primary central nervous system lymphoma.

## Data Availability

The data used to support the findings of this study are available from the corresponding author upon request.
